# Clinical Demonstration of Dr. Bonwill's Methods of Practice

**Published:** 1900-03

**Authors:** Geo. V. L. Brown


ARTICLE VII.

### CLINICAL DEMONSTRATION OF DR. BONWILL'S METHODS OF PRACTICE.

REPORTED BY GEO. V. L. BROWN, B. A., D. D. S., M. D., C. M.

The following report of a clinical exhibition of patients, operations and methods, given by Dr. W. G. A. Bonwill, in his office in Philadelphia, June, 1897, before a committee appointed by the Section of Stomatology of the American Medical Association, the substance of which, though not properly within the limits of the purpose for which the section was organized, and therefore ineligible for publication in the columns of the official journal of the American Medical Association, is, nevertheless, so full of matter of such great importance to the dental profession that it is hoped its publication at this time, and in the manner contemplated, may lead to the preservation, with credit to their originator, of the methods described.

The committee were shown some forty patients. Work was thoroughly examined, and all were interrogated with regard to the length of time since the fillings had been inserted, the number of years they had been under Dr. Bonwill's care, and their opinions generally. These questions, as every one will know who remembers Dr. Bonwill's almost absolute deafness, could be frankly answered without fear of being overheard by Dr. Bonwill. There was, therefore, no possible reason why the most perfect truth should not have been told. Such a demonstration each of us felt it had never been his privilege to witness before and doubtless never would again.

One or two of the oldest had been under Dr. Bonwill's care for some forty odd years; many more than thirty years; others a shorter period, and some were very young patients. To one who had been accustomed to see Dr. Bonwill operating under the disadvantages of large public clinics, where everything possible constantly occurred to increase his natural nervousness, the beautiful work, with its careful polish and the exactness of everything in his private office was a revelation, when compared with the opinion too often expressed, that his work was rough and crude. Nothing could be further from the truth. Down to the smallest detail, all was neatness, exactness and perfection. The impression was a profound one, and doubtless many years of development will be required before we can fully comprehend the merit and value which underlay that which we only saw upon the surface.

As everyone knows, Dr. Bonwill bound his faith to Abbey's gold foil, claiming for it properties which other kinds did not possess, and his fillings, made as he told us, with this foil, would aggregate a large number, and if it were possible to roll them all together would make a sheet of gold marvelous when considered as having been put together, piece by piece, by one man.

The cavity outlines were quite marked by contrast to the square, more box-like forms advocated by many teachers at the present time. In his work every corner was rounded, every outline a perfect curve, but the boldness with which cavity walls had been cut away on the lingual and buccal margins, showed that many years ago he had anticipated, in its fullest extent, the value of extension for prevention. The contour of the filling in each case was bold and clearly marked, points of contact showing a careful consideration of most favorable adjustment.

The plus contours shown were quite astonishing (cantilever size, the author called them), many cases showing that, where teeth had been lost in early life, and crowding together had narrowed the space to some extent, but had caused a tipping forward, particularly of the molar teeth, endangering the future usefulness of the tooth through mal-occlusion, with a boldness characteristic of the man, he had not hesitated to extend his gold forward in a rounded, tapering form, something after the manner of the horn of a blacksmith's anvil until the point rested securely in contact with the distal surface of the tooth in front. Only those familiar with the peculiar properties of gold and the difficulty of perfect condensation of an over-hanging mass in such form as to give secure resistance in all directions, can appreciate the triumph of the success this work meant. As for the value of supporting leaning teeth in that manner, and its import upon the future usefulness and long retention of the teeth, I think we have all of us yet much to learn before this can be fully appreciated.

The occlusal surfaces in many instances showed an attempt, at least, to reproduce angles and cusp lines rather than the smooth concavity with which we are, as a rule, familiar. The broadly over-lapping margins, both of gold and amalgam fillings, successfully carried out the design of clasping and supporting the frail walls of teeth rather than to depend solely upon support of the filling by the walls, and were a revelation to any one who had been led to believe that alloy fillings could not be relied upon for this purpose, by reason of insufficient edge strength.

The committee was not given, as fully as might have been, the nature and manner of working these alloys, too much of other things crowding out its minute consideration.

In many teeth gold and alloy were mixed in the same tooth. In almost every instance the color of the alloy had remained untarnished after the first evidently careful polishing, and each, of all these fillings, whether gold, or amalgam, or both, seemed to be doing service with perfect satisfaction. Amalgam seemed to last and protect against caries as well as gold, and vice versa, the lesson of it all being borne strongly in upon us that, beyond and above the details, which we all recognize as important in filling of teeth, there was a something in the work that this man did which rendered it capable of immunizing that particular portion of the mouth against the inroad of bacteria.

Many patients whose teeth showed that at some previous time there had been marked tendency to caries, reported having had almost no fillings placed in their teeth since the completion of the first general work, after coming under Dr. Bonwill's hands. The result certainly justified the means in every particular.

Pink gutta-percha fillings packed securely in proximo-occlusal cavities, extending across the interdental space to the next tooth, were to be seen in a number of mouths. These, Dr. Bonwill explained, were inserted for the purpose of effecting a slow but perfect separation. He left them about one year, after which, sufficient space was gained to admit of the perfect contouring of the fillings and the sensitive dentin lost much or all of its sensibility, so that the excavation for the gold or alloy fillings gave little or no pain, and there was slight danger of exposing pulps, a thing which seldom occurred in his practice.

Dr. Bonwill had great faith in gutta-percha as a stopping for children's teeth, and for use as just described. He claimed that the slow separation it effected allowed the adjustment of the tooth moved in its relation with the opposing one in the jaw and prevented the likelihood of future trouble from mal-occlusion, particularly in regard to pyorrhea alveolaris. Among all those cases none were found to have active symptoms of pyorrhœa, but several showed by the denuded surfaces of the roots and recessions of the gums, that they had been so affected, yet none were found to be noticeably loose, and there was an entire absence of discharge.

Very few crowns were seen, the extensive contour operations seeming to have reduced the necessity for their use to a minimum. Few artificial dentures were shown, such as were, being partial and having clasps for the natural teeth. The method of adjusting these clasps, as also Dr. Bonwill's now famous methods of articulation and arrangement of the teeth in the arch, according to his geometrical principles, are all fully set forth in his various writings, and need only mention here in that they seemed to be fully equal to the praise that their author himself has bestowed upon them.

His views on cataphoresis or the plain current of electricity are better set forth in the paper read before the Section of Stomatology of the American Medical Association, at its meeting held in Philadelphia in June, 1897, than would be possible in any report the committee could make, particularly since no demonstrations of this sort were shown, as it had been expected there would be; nor were any demonstrations given of rapid breathing as a means of performing painless operations by diversion of the will, but the writer can fully testify to the fact that nearly twenty years ago he heard Dr. Bonwill explain the use of this method in performing minor surgical operations, and its constant use during some few years past has absolutely demonstrated that an extremely valuable truth underlies this apparently simple experiment.

No fistulous openings were observed in any of the mouths; the gums and mucous membrane in all cases seemed to be in such healthful condition as would naturally be expected with those who observed the minute instructions given them.

In his own peculiar, very peculiar, way, one might say Dr. Bonwill seemed to possess, not only the absolute confidence of his patients but also their simple and almost blind obedience to all his wishes, they submitting, apparently as a matter of course, to his most arbitary exactions.

Some patients were shown for whom, in their earlier years, the method advocated by Dr. Bonwill, of cutting V-shaped spaces upon proximate surfaces of the anterior teeth, with little noticeable change upon the labial aspect, but quite sharply cut away upon the lingual side, in order to facilitate self-cleaning with a view to the prevention of caries, had been practiced. So far as the result was concerned, he seemed to be highly successful in accomplishing that which he desired.

In Dr. Bonwill's own words, it was a lesson of forty-two years' work, and in answer to his question whether it showed the hand of art or mechanism, we would unreservedly say that in a high degree of art and mechanism paid silent tribute to the wenderful genius, through the instrumentality of which so much had been accomplished in the saving of human teeth, giving, as it did, one man the power to exert an influence upon human life far beyond the possibility of measurement by present standards.—*Dental Brief.*

